# Thermodynamic properties of water molecules in the presence of cosolute depend on DNA structure: a study using grid inhomogeneous solvation theory

**DOI:** 10.1093/nar/gkv1133

**Published:** 2015-11-03

**Authors:** Miki Nakano, Hisae Tateishi-Karimata, Shigenori Tanaka, Florence Tama, Osamu Miyashita, Shu-ichi Nakano, Naoki Sugimoto

**Affiliations:** 1 Frontier Institute for Biomolecular Engineering Research (FIBER), Konan University, 7-1-20 Minatojima-minamimachi, Chuo-ku, Kobe 650-0047, Japan; 2 Advanced Institute for Computational Sciences, RIKEN, 7-1-26, Minatojima-minamimachi, Chuo-ku, Kobe 650-0047, Japan; 3 Department of Computational Science, Graduate School of System Informatics, Kobe University, 1-1, Rokkodai, Nada-ku, Kobe 657-8501, Japan; 4 Department of Physics, Graduate School of Science, Nagoya University, Furo-cho, Chikusa-ku, Nagoya 464-8602, Japan; 5 Faculty of Frontiers of Innovative Research in Science and Technology (FIRST), Konan University, 7-1-20, Minatojima-minamimachi, Chuo-ku, Kobe 650-0047, Japan; 6 Graduate School of Frontiers of Innovative Research in Science and Technology (FIRST), Konan University, 7-1-20 Minatojima-minamimachi, Chuo-ku, Kobe 650-0047, Japan

## Abstract

In conditions that mimic those of the living cell, where various biomolecules and other components are present, DNA strands can adopt many structures in addition to the canonical B-form duplex. Previous studies in the presence of cosolutes that induce molecular crowding showed that thermal stabilities of DNA structures are associated with the properties of the water molecules around the DNAs. To understand how cosolutes, such as ethylene glycol, affect the thermal stability of DNA structures, we investigated the thermodynamic properties of water molecules around a hairpin duplex and a G-quadruplex using grid inhomogeneous solvation theory (GIST) with or without cosolutes. Our analysis indicated that (i) cosolutes increased the free energy of water molecules around DNA by disrupting water–water interactions, (ii) ethylene glycol more effectively disrupted water–water interactions around Watson–Crick base pairs than those around G-quartets or non-paired bases, (iii) due to the negative electrostatic potential there was a thicker hydration shell around G-quartets than around Watson–Crick-paired bases. Our findings suggest that the thermal stability of the hydration shell around DNAs is one factor that affects the thermal stabilities of DNA structures under the crowding conditions.

## INTRODUCTION

Depending on sequence and solution conditions, nucleic acids can adopt triplex, i-motif and G-quadruplex structures in addition to the canonical B-form duplex composed of Watson–Crick base pairs (1–4). To date, more than ten different nucleic acid structures have been characterized through biophysical and biochemical studies, and the roles of these structures in biological functions have begun to be elucidated (1–4). The thermal stabilities of these nucleic acid structures will determine whether structures impact biological processes such as transcription, translation and interactions with other biomolecules in living cells (5–7). For example, age-related diseases and premature aging syndromes are characterized by short telomeres, which form G-quadruplex *in vivo* (8–11). Some proteins, such as the tumor suppressor protein p53 and Human DNA polymerase-_L_, have evolved to recognize certain Watson–Crick or Hoogsteen base pairs (12–14). In addition, there is evidence that DNA strands like those observed in triplet repeat regions of the *frataxin* gene form triplex structures that inhibit gene expression (15). To understand physiology and metabolism *in vivo*, it is necessary to understand the principles that govern thermal stability of nucleic acid structures in conditions that mimic those of living cells.

Living cells contain high concentrations of biomolecules and soluble and insoluble components resulting in a condition referred to as molecular crowding (16–21). Under molecular crowding condition, hydration properties around biomolecules differ from those in dilute solution due to fewer free water molecules and altered dielectric constant and viscosity (16–21). Molecular crowding conditions impact the thermal stabilities of DNA structures in a manner dependent on the type of DNA structure (22–29). Formation of nucleic acid structures is accompanied by the formation of a hydrogen-bonded network of water molecules that surround the nucleic acid surface (30–33). These water networks are highly sensitive to the water activity in the solution and depend on the type of DNA structure formed. For example, ethylene glycol, polyethylene glycol, polyols and polysaccharides decrease the thermal stability of long and short DNA duplexes, whereas the formation of triplexes, G-quadruplexes, i-motifs and Holiday junctions are stabilized in the presence of cosolutes (26–28).

In general, the water activity is decreased by the presence of cosolutes (16,17,34). It is thought that decline in water activity is unfavorable for formation of Watson–Crick base pairs, a reaction that requires uptake of water molecules, and is favorable for formation of G-quartets, which is accompanied by the release of water (23,27–29,33–37). However, this interpretation, which associates the change in equilibrium between folded and unfolded DNA structures under molecular crowding conditions with the number of waters incorporated into DNA strands conflicts with the microscopic picture that the solvent accessible surface area around Watson–Crick base pairs is smaller than the sum of the areas around the isolated bases (38).

To investigate the equilibrium state of DNA structures in an aqueous environment, the free energy of the entire system must be considered, including both solute and solvent. Recently, a new method for analysis of hydration properties of biomolecules called grid inhomogeneous solvation theory (GIST) has been developed by Gilson *et al*. (39). In GIST, the spatial integrals in inhomogeneous fluid solvation theory are replaced by discrete sums, called voxels, of the boxes of a three-dimensional grid. Snapshots from explicit solvent simulations are used to estimate localized solvation entropies, solvation interaction energies and free energies of the water molecules associated with the voxels. Summation of these thermodynamic quantities over voxels yields information about hydration thermodynamics. Use of this method allows visualization of the spatial distribution of the thermodynamic properties of water molecules around biomolecules. Park *et al*. investigated the effects of thermodynamics of the solvent on the structural stability of RNA composed of AUUCU repeats, which causes Spinocerebellar Ataxia Type 10. They showed that water molecules coordinate potential hydrogen bonds between atoms of RNA (40).

In this study, we investigated hydration properties around a DNA that adopts a hairpin (HP) loop closed by Watson–Crick base pairs and a DNA that adopts a G-quadruplex structure using GIST. We compared the free energies of water molecules around these DNA structures under the different ethylene glycol (EG) concentrations. We found that the free energy of the water molecules around DNAs was increased with increasing EG concentration due to the reduction of water–water interactions. The interactions among waters surrounding Watson–Crick base pairs were more disturbed by EG than were those around the unfolded DNA structure, contributing to the destabilization of the hairpin structure in EG compared to buffer alone. The negative electrostatic potential around G-quartets resulted in formation of thicker hydration shell around the G-quadruplex than around the duplex region; the water–water interactions around a G-quadruplex had more robustness against disruption by the cosolutes. Our findings indicate that the water–water interactions around DNA depend on cosolutes and contribute to the thermal stabilities of DNA structures.

## MATERIALS AND METHODS

### Grid inhomogeneous solvation theory (GIST)

GIST was developed by Gilson *et al*. (39). In GIST, the spatial integrals used in inhomogeneous solvation theory (41,42) are replaced by discrete sums over the voxels of a three-dimensional grid, where the quantities on the grid are computed from the stored frames of a molecular dynamics (MD) simulation. In GIST, relative water density in voxel *k* to that of bulk water, *g*(r*_k_*), is defined as follows:

(1) \begin{equation*} g({\bf r}_k ) \equiv \frac{{\rho ({\bf r}_k )}}{{\rho ^0 }} = \frac{{n({\bf r}_k )}}{{\rho ^0 V_k }}. \end{equation*}

Here, *k* is the voxel index, and **r***_k_* is the center position of voxel *k*. *ρ^0^* is the number density of bulk waters, and *ρ*(**r***_k_*) is the number density of water molecules in voxel *k*. *V_k_* is the volume of voxel *k*, and *n*(**r***_k_*) is the number of waters within voxel *k* averaged across all frames used for GIST analysis. In this calculation, a water molecule is considered to lie in voxel *k* if its oxygen atom is in the voxel, and *g*(**r***_k_*) is assumed to be uniform within each voxel. Using *g*(**r***_k_*) and force field parameters employed for the MD simulation, for the water molecules in the voxel *k*, the translational and orientational entropies, *S_tr_*(**r***_k_*) and *S_or_*(**r***_k_*), and the interaction energies (including electrostatic and van der Waals interactions) among the water molecules in voxel *k* and solute or other water molecules, *E_sw_*(**r***_k_*) or *E_ww_*(**r***_k_*), are calculated for each voxel. Solvation entropies and solvation interaction energies in the voxel *k* are calculated by subtracting the entropies and interaction energies for bulk water from those for the water molecules in the voxel *k*, defined as *ΔS_tr_*(**r***_k_*), *ΔS_or_*(**r***_k_*), *ΔE_sw_*(**r***_k_*) and *ΔE_ww_*(**r***_k_*). These calculations are described in detail in the original paper (39).

To compare these thermodynamic parameters among different systems or different regions, normalized thermodynamic parameters were defined as follows:

(2) \begin{equation*} T\Delta S_{tr}^w (R) = \frac{1}{{n(R)}}T\Delta S_{tr} (R) = \frac{1}{{n(R)}}\sum\limits_{k \in R} {T\Delta S_{tr} ({\bf r}_k )} , \end{equation*}

(3) \begin{equation*} T\Delta S_{or}^w (R) = \frac{1}{{n(R)}}T\Delta S_{or} (R) = \frac{1}{{n(R)}}\sum\limits_{k \in R} {T\Delta S_{or} ({\bf r}_k )} , \end{equation*}

(4) \begin{equation*} \Delta E_{sw}^w (R) = \frac{1}{{n(R)}}\Delta E_{sw} (R) = \frac{1}{{n(R)}}\sum\limits_{k \in R} {\Delta E_{sw} ({\bf r}_k )} , \end{equation*}

(5) \begin{equation*} \begin{array}{*{20}c} {\Delta E_{ww}^w (R) = \frac{1}{{n(R)}}\Delta E_{ww} (R)} \\ { = \frac{1}{{n(R)}}\left( {E_{ww} (R) - E_{ww}^0 (R)} \right)} \\ { = \frac{1}{{n(R)}}\left( {\sum\limits_{k \in R} {E_{ww} ({\bf r}_k )} - \rho ^0 V(R)\varepsilon _{ww}^0 } \right)} \\ { = \frac{1}{{n(R)}}\left( {\sum\limits_{k \in R} {E_{ww} ({\bf r}_k )} - \frac{{\rho ^0 }}{{\rho (R)}}n(R)\varepsilon _{ww}^0 } \right)} \\ \end{array}, \end{equation*}

(6) \begin{equation*} V(R) = \sum\limits_{k \in R} {V_k } , \end{equation*}

(7) \begin{equation*} n(R) = \sum\limits_{k \in R} {n({\bf r}_k )} , \end{equation*}

(8) \begin{equation*} \rho (R) = \frac{{n(R)}}{{V(R)}}, \end{equation*}

where *T* is the absolute temperature and *ε_ww_^0^* is the mean water–water interaction energy for bulk water. *R* is the region specified for GIST analysis in the simulation system. *V*(*R*) is the sum of volume for the voxels included in the region *R*, *n*(*R*) is the average number of water molecules in the region *R* across all frames and *ρ*(*R*) is the number density of water molecules in the region *R*. In the original paper (39), the water–water interaction energy for bulk water in the region *R*, *E_ww_^0^*(*R*) was defined as *n*(*R*)*ε_ww_^0^*. With this definition, the density of water in region *R*, *ρ*(*R*) = *n*(*R*) / *V*(*R*), can differ from the true density of bulk water, *ρ^0^*. Using the definition of the original paper, *ΔE_ww_*(*R*) is underestimated in the case of *ρ*(*R*) > *ρ^0^*, whereas *ΔE_ww_*(*R*) is overestimated in the case of *ρ*(*R*) < *ρ^0^*. Therefore, in the present study, we redefined *E_ww_^0^*(*R*) as $\rho ^0 V(R)\varepsilon _{ww}^0 = \frac{{\rho ^0 }}{{\rho (R)}}n(R)\varepsilon _{ww}^0$, as shown in Equation (5).

Finally, the difference of normalized average free energy of one water molecule relative to bulk water in the region *R* can also be written as:

(9) \begin{eqnarray*} &&\Delta G^w (R) =\nonumber \\ &&- T\Delta S_{tr}^w (R) - T\Delta S_{or}^w (R) + \Delta E_{sw}^w (R) + \Delta E_{ww}^w (R). \end{eqnarray*}

As shown in Equation (9), *ΔE_sw_^w^*(*R*) + *ΔE_ww_^w^*(*R*) corresponds to the difference in enthalpy between a water molecule in the region *R* and that in the bulk water.

### Materials

**Figure 1.**
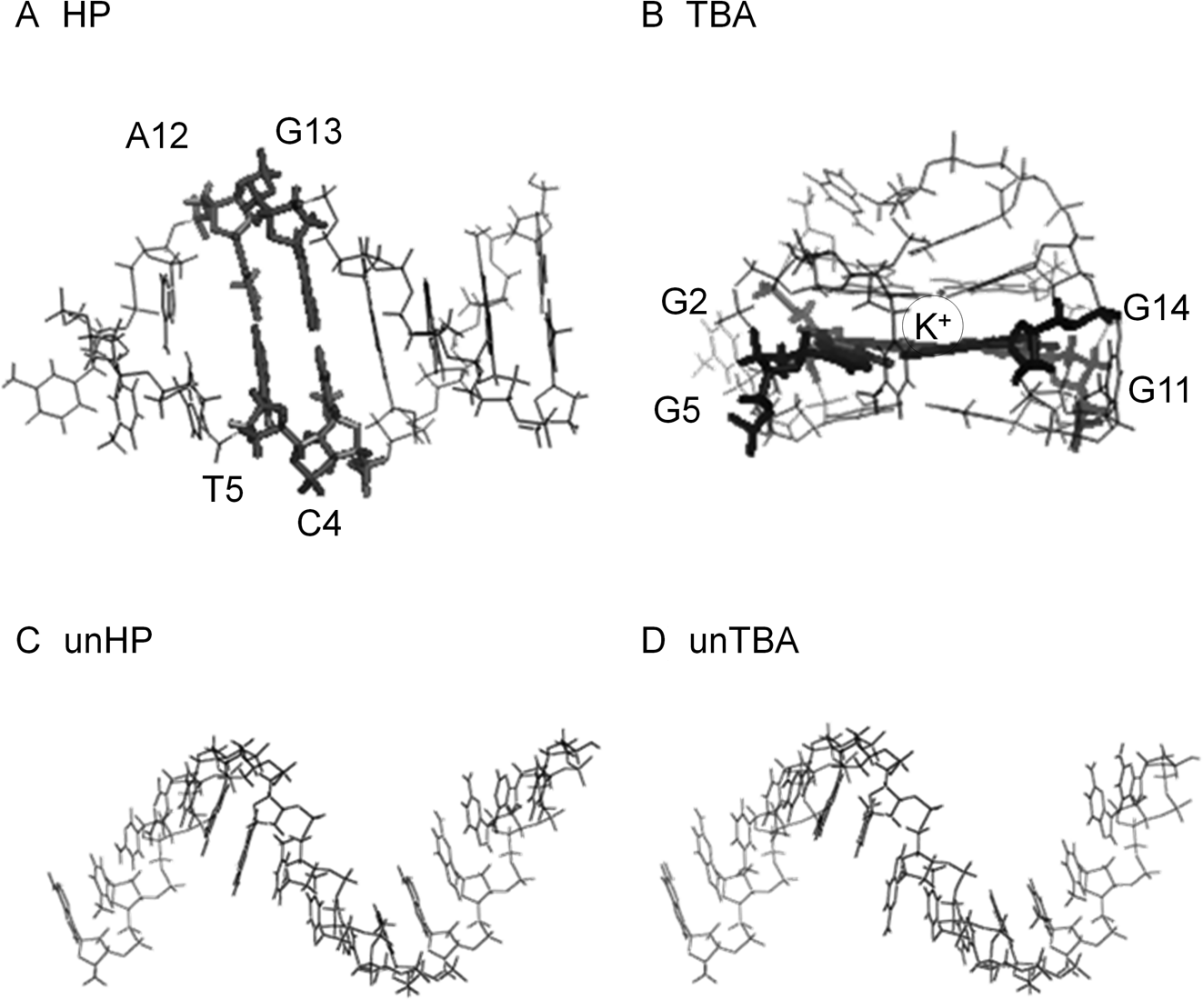
Structures of (**A**) HP, (**B**) TBA, (**C**) unHP and (**D**) unTBA. In panel B, circle represents K^+^ ion at the center of the molecule, which was constrained to the position indicated in PDB (1C35) during the molecular dynamics (MD) simulation. In panels A and B, the locations of Watson–Crick base pairs and G-quartet used for the local hydration analysis are represented with thick lines and labeled in the whole structure (in Section 4: referred to as WC in HP and as G4 in TBA).

To investigate the hydration properties of Watson–Crick base pairs and G-quartets with Hoogsteen base pairs, we chose two DNA structures registered in Protein Data Bank (PDB) as 1AC7 (43) and 1C35 (44). 1AC7 is a 16-mer of sequence 5′-ATCCTAGTTATAGGAT-3′ (loop region underlined), which forms a hairpin (HP) with a stem of six Watson–Crick base pairs. 1C35 is a 15-mer, 5′-GGTTGGTGTGGTTGG-3′, which forms a G-quadruplex known as the thrombin binding aptamer (TBA), which folds into a G-quadruplex stabilized by two guanine quartets (G-quartets). 1AC7 and 1C35 have been used in many previous molecular dynamics simulation studies (45–50). For comparison of the hydration properties of folded and unfolded DNA structures, we constructed B-form duplexes of the sequences 1AC7 and 1C35 using Discovery Studio 3.5 (51) and then removed the complementary strands. The single strand in the B-form was then used as the ‘unfolded’ structures unHP and unTBA. It should be noted that a single-stranded DNA has considerable flexibility in an aqueous environment (52–55); however, it is impossible to take into account all the potential structures in a molecular dynamics simulation. Initial structures of the HP, TBA, unHP and unTBA are shown in Figure 1A–D.

### Molecular dynamics simulations

In this study, we used EG as the water-soluble neutral cosolute. We performed MD simulations with explicit water molecules and different EG concentrations (0.0 M, 2.5 M and 5.0 M) for each DNA structure. The thermal stabilities of DNA structures have been analyzed in the presence of EG (23,27,28). EG decreases the melting temperatures of Watson–Crick duplexes and increases those of G-quadruplexes (23,27). We placed DNA, EG, K^+^ and water molecules in the simulation box using the Packmol program (56). To know the properties of waters around the stable G-quadruplex structure, the position of potassium ion located at the center of TBA in PDB structure (1C35) was constrained during the MD simulations, because it is well known that G-quadruplex structure of TBA is stable if the cation is located at the center of the molecule (49,50). After equilibration, the simulation box sizes were 70 Å^3^ for HP and TBA, and 75 Å^3^ for unHP and unTBA to provide sufficient hydration space around DNAs. We then constructed the initial coordinate and force field parameter files for the MD simulations using the leap module in AMBER 14 (57). The AMBER force field ff99bsc0 was applied to DNA (58), and TIP3P model was applied to water molecules (59). The force field parameters of EG were generated using antechamber and gaff modules included in AMBER Tools 14 (60,61). All atomic charges were determined through standard RESP/6–31G(d) calculations (62), and the charges for two oxygen atoms in the phosphate group are set to be the same (58). The system names and components used in our MD calculations are listed in Table 1.

**Table 1.** List of the system names, structures and components for the molecular dynamics simulations

		Number of					
Name	structure(PDB ID)	DNA strands	DNA bases	K^+(*a*)^	water	EG	EG concentration [mol/l]
HP	1AC7	1	16	15	11 109	0	0.0
					11 505	600	2.5
					9 507	1200	5.0
TBA	1C35	1	15	14	11 119	0	0.0
					11 499	600	2.5
					9 601	1200	5.0
unHP	unfolded DNA	1	16	15	11 118	0	0.0
					11 480	600	2.5
					9 523	1200	5.0
unTBA	unfolded DNA	1	15	14	11 134	0	0.0
					11 507	600	2.5
					9 591	1200	5.0

^a^Number of potassium ions added to neutralize the simulation system.

MD simulations were performed with AMBER14 software package (57). The optimization of water molecules, ions and EG molecules was carried out in 1000 steps with the structure of DNA fixed. Next, the system was heated to 298 K for 100 ps. After that, MD simulations were carried out in the NPT ensemble at 1 atm and 298 K for 100 ns with 2 fs simulation time steps. Throughout the MD simulations, periodic boundary conditions were employed and all electrostatic interactions were calculated using the particle-mesh Ewald (PME) method. An 8.0 Å cutoff length was used to calculate the direct space sum of PME, and lengths of bonds involving hydrogen atoms were constrained using the SHAKE algorithm (63). To obtain information about the structure and thermodynamics of the water molecules around the specified DNA structure, DNA structures were constrained during the MD simulations. In this study, we used last 50 ns trajectories for our GIST analyses, where the numbers of water or EG molecules around DNA were fully equilibrated (Supplementary Figure S1). Calculations were performed on the SGI UV 1000 system at the Research Center for Computational Science, Okazaki and on DELL Precision T5400 system at Kobe University.

### Analysis of MD simulation data

**Figure 2.**
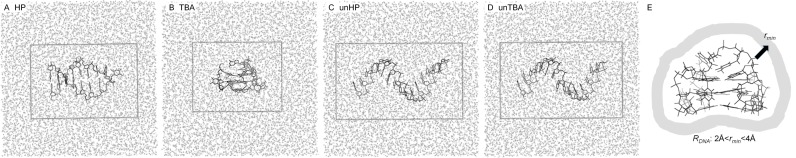
Whole MD systems and grid inhomogeneous solvation theory (GIST) analysis regions for (**A**) HP, (**B**) TBA, (**C**) unHP and (**D**) unTBA, and (**E**) the definition of GIST analysis *R_DNA_* used in this study. DNA strands are shown in black stick models, and oxygen atoms of water molecules are shown as gray dots. Rectangular boxes in panels A, B, C and D represent the GIST analysis regions for each system. In panel 2E, *r_min_* is the minimum distance between DNA atom and the center of voxel, and *R_DNA_* (colored in gray around DNA) is the GIST analysis region in this study.

To investigate the thermodynamic parameters of the water molecules around DNA, we used the GIST program included in cpptraj module in AMBER 14 (64). We set the GIST grid size to 0.5 Å^3^, and set the GIST analysis region to cover the whole DNA structure with a margin of at least 7 Å. The box sizes were 36 × 34 × 49 Å^3^ for HP, 39 × 32 × 41 Å^3^ for TBA, 35 × 35 × 58 Å^3^ for unHP and 35 × 35 × 56 Å^3^ for unTBA, respectively. Locations of GIST analysis regions relative to the MD systems are shown in Figure 2A–D. In this study, as the first step to investigate the effects of environment on the thermal stabilities of DNA structures, we focused on the thermodynamic properties of water molecules around DNA, although it is expected that the interaction between cosolutes and DNA is also important. We removed all potassium ions and EG molecules from the simulation boxes before applying GIST analysis because GIST assumes that the solute of interest comprises all molecules in the simulation box except for water molecules.

In this study, we set the number density, *ρ*^0^, to 0.0329 Å^−3^, and the mean water–water interaction energy, *ε_ww_^0^*, to −9.565 kcal/mol per water molecule as the reference values for bulk water. These values were obtained from preliminary MD simulation with only TIP3P water molecules. To study the hydration properties of DNA, we defined the minimum distance between DNA atoms and the center of voxel as *r_min_*, and studied the voxels with 2 Å < *r_min_* < 4 Å defined as region *R_DNA_*. This region was considered as the first hydration shell around the DNA as no significant differences in water densities outside this area were observed among different DNA structures (see Results section). We obtained the thermodynamic parameters for a water molecule by normalizing the sum of each parameter for the voxels in the region *R_DNA_* by the number of water molecules in the same region. An illustration of the GIST region *R_DNA_* is shown in Figure 2E. To evaluate the convergence of thermodynamic parameters, we calculated the thermodynamic parameters normalized by the number of water molecules in the region *R_DNA_* for TBA for 5.0 M EG concentration using different numbers of frames. As shown in Supplementary Figure S2A, solvation interaction energies, *ΔE_sw_^w^*(*R_DNA_*) and *ΔE_ww_^w^*(*R_DNA_*), were sufficiently converged with 10 000 frames (corresponding to 10 ns trajectories). In contrast, as shown in Supplementary Figure S2B, convergence of solvation entropy terms, *TΔS_tr_^w^*(*R_DNA_*) and *TΔS_or_^w^*(*R_DNA_*), was slower than that of solvation interaction energies, and the orientational entropy term was not converged with 50 000 frames. Absolute values for the solvation entropy terms were about one-tenth of the values of the solvation interaction energies, and the differences of solvation entropy terms calculated with between 40 000 and 50 000 frames were less than those of solvation interaction energies. Therefore, we used 50 000 frames obtained from 50–100 ns MD trajectories for GIST analyses for each system.

## RESULTS

### Water and EG densities around DNA

To estimate general hydration properties around DNAs, we calculated the density profiles of solvent (water) and cosolute (EG) molecules around DNAs. The number density of molecules, *ρ_molecule_*(*r_min_*) was calculated as follows:

(10) \begin{equation*} \rho _{molecule} (r_{min} ) = \left\langle {\frac{{n_{molecule} ({\bf r}_k )}}{{V_k }}} \right\rangle _{r_{min} \le {\bf r}_k < r_{min} + \Delta r}. \end{equation*}

*r_min_* is the minimum distance between DNA atoms and the center of voxel, and *Δr* was set to 0.5 Å (39). *k* is the voxel index, and **r***_k_* is the center position of voxel *k*. *V_k_* is the volume of voxel *k*, and *n_molecule_*(**r***_k_*) is the number of molecules within voxel *k* averaged across all frames used for GIST analysis.

**Figure 3.**
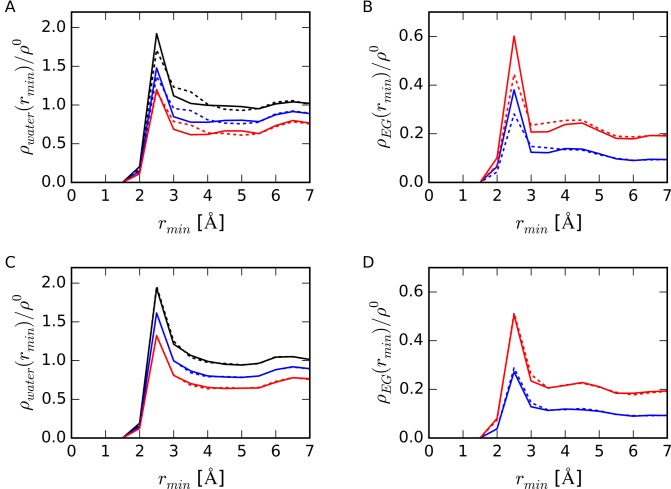
(**A**) Densities of water molecules near DNA strands relative to bulk water density, *ρ_water_*(*r_min_*)/*ρ*^0^ and (**B**) relative number densities of EG molecule to bulk water, *ρ_EG_*(*r_min_*)/*ρ*^0^, around HP (solid lines) and TBA (dotted lines). (**C**) *ρ_water_*(*r_min_*)/*ρ*^0^ and (**D**) *ρ_EG_*(*r_min_*)/*ρ*^0^ around unHP (solid lines) and unTBA (dotted lines). Bulk water density (*ρ*^0^) was set to 0.0329 Å^−3^. Black, blue and red lines represent data from 0.0 M, 2.5 M and 5.0 M EG concentrations, respectively.

Figure 3 shows the relative densities of water and of EG to bulk water density, *ρ_water_*(*r_min_*) / *ρ*^0^ and *ρ_EG_*(*r_min_*) / *ρ*^0^, respectively, around folded and unfolded DNAs at different EG concentrations. As shown in Figure 3A and B, in the region 3.0 Å < *r_min_* < 4.0 Å, both *ρ_water_*(*r_min_*) and *ρ_EG_*(*r_min_*) around folded TBA were higher than those around folded HP, although the peak heights at *r_min_* of 2.5 Å for HP were higher than those for TBA. The profile of the density distributions around HP structure was sharp, whereas that for TBA was broad and the TBA hydration shell was thicker than that of HP. There were no significant differences in density profiles between unHP and unTBA as shown in Figure 3C and D. These results indicate that the differences in density profiles around structured HP and TBA were caused by the difference in DNA structures rather than the difference of DNA sequences.

Around all DNA structures, the peak height of *ρ_water_* decreased as the concentration of EG increased, whereas the height of *ρ_EG_* increased. At 5.0 M EG concentration, the peak heights of *ρ_EG_* / *ρ*^0^(*r_min_* 2.5 Å) were 0.60 for HP, 0.44 for TBA, 0.51 for unHP and 0.51 for unTBA. This peak height for HP was larger than that for unHP, whereas that for TBA was smaller than that for unTBA. Figure 3B and D showed that accessibility for EG molecules to DNA depended on the DNA structure. Molecules of EG contacted the structured HP but did not directly contact TBA.

### Thermodynamic parameters for water molecule in the first hydration shell around DNA

**Figure 4.**
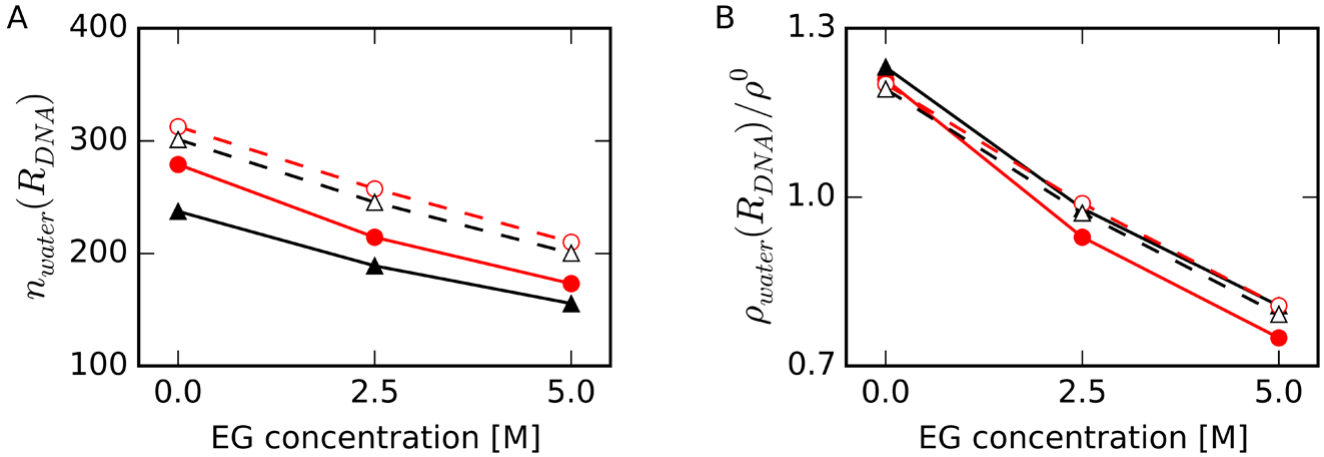
(**A**) Number of water molecules in the region *R_DNA_*, *n_water_*(*R_DNA_*), and (**B**) relative number density of water molecules in the region *R_DNA_* to the density of bulk water, *ρ_water_*(*R_DNA_*) / *ρ*^0^, at different EG concentrations for HP (filled red circles with solid lines), TBA (filled black triangles with solid lines), unHP (open red circles with dashed lines) and unTBA (open black triangles with dashed lines). Standard errors of the mean (SEM), which were calculated with the average values for every 10 ns data from 50 ns to 100 ns MD simulations, are enough small within the sizes of symbols (also in Figures 5–9).

To investigate the solvent properties near DNAs, we focused on the water molecules in the first hydration shell around DNA. From the results of water and EG density profiles as shown in Figure 3, we defined the region within 2.0 Å < *r_min_* < 4.0 Å as the first hydration shell around DNA, region *R_DNA_*, and calculated thermodynamic parameters for a water molecule in this region. No significant differences in water densities outside this area were observed. Figure 4A shows the number of water molecules, *n_water_*(*R_DNA_*), and Figure 4B shows the averaged relative number density of water molecules, *ρ_water_*(*R_DNA_*) */ ρ^0^*, in the region *R_DNA_*. For both HP and TBA, *n_water_*(*R_DNA_*) values around the folded structures were smaller than those around the unfolded structures. These results show that dehydration occurred upon formation of base pairs and base quartets from the unfolded structures. The difference in *n_water_*(*R_DNA_*) for TBA and unTBA was larger than that for HP and unHP for all EG concentrations, whereas *ρ_water_*(*R_DNA_*) around the DNA in the absence of EG were almost the same (within 3%) for all DNA structures. The decreases in *n_water_*(*R_DNA_*) and *ρ_water_*(*R_DNA_*) */ ρ^0^* for HP upon addition of EG were larger than those for TBA, unHP and unTBA. These results were consistent with the result shown in Figure 3B: EG replaced water molecules around HP more readily than it did water molecules around TBA.

**Figure 5.**
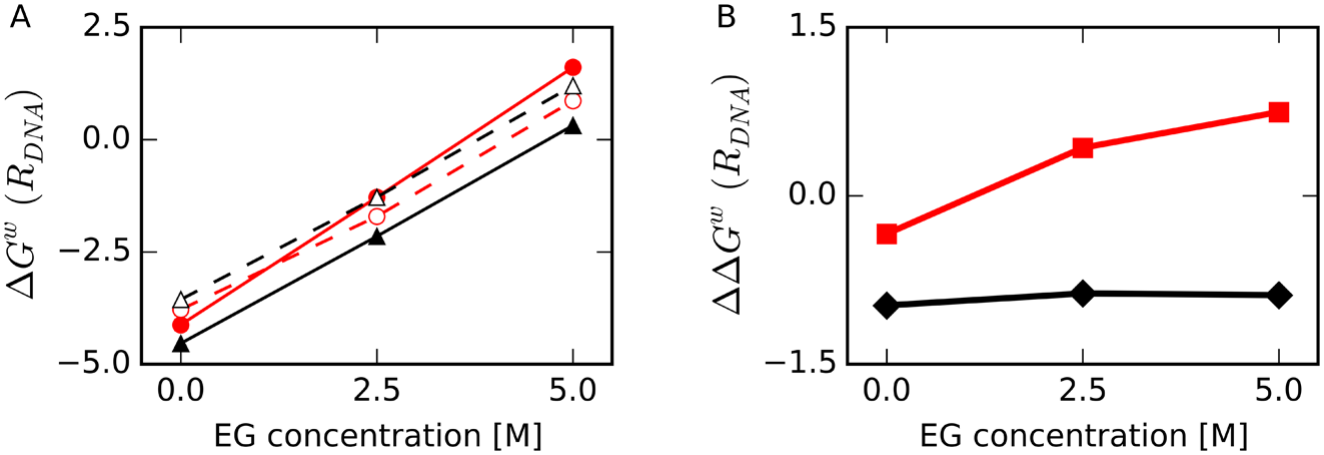
(**A**) Normalized average free energy of a water molecule relative to bulk water in the first hydration shell around DNA, *ΔG^w^*(*R_DNA_*), as a function of EG concentration for HP (filled red circles with solid line), TBA (filled black triangles with solid line), unHP (open red circles with dashed line) and unTBA (open black triangles with dashed line). (**B**) Difference in *ΔG^w^*(*R_DNA_*) between folded and unfolded structures, *ΔΔG^w^*(*R_DNA_*), for HP versus unHP (red squares) and TBA versus unTBA (black diamonds). All energies are shown in kcal/mol/water at 298 K.

We calculated the thermodynamic energy of water molecules normalized by the average number of water molecules in the region *R_DNA_* at 298 K, which was the temperature used in our MD simulations (Figure 5A). In the presence of EG, *ΔG^w^*(*R_DNA_*) was higher for all the structures than in the absence of EG. Because *ρ_water_*(*R_DNA_*) decreased with increasing EG concentration as shown in Figure 4B, these results were reasonable under the assumption that *ρ_water_*(*R_DNA_*) reflects the probability of finding a water molecule in *R_DNA_* in terms of potential of mean force, where *ΔG^w^*(*R_DNA_*) was proportional to –ln(*ρ_water_*(*R_DNA_*) / *ρ*^0^) (65).

As illustrated in Figure 5A, the increase in *ΔG^w^*(*R_DNA_*) for HP was larger for other structures. Figure 5B shows the normalized free energy difference of a water molecule in the region *R_DNA_* between folded and unfolded structures, where *ΔΔG^w^*(*R_DNA_*) = *ΔG^w^*(*R_DNA_*, fold) – *ΔG^w^*(*R_DNA_*, unfold). In the absence of EG, *ΔG^w^*(*R_DNA_*) values for folded HP and TBA structures were lower than those of unfolded structures. At the highest EG concentration, *ΔG^w^*(*R_DNA_*) for HP was higher than that for unHP. This result suggests that free energies of water molecules around HP contribute to destabilization of the HP structure at high EG concentration. In contrast, the relative thermal stability of folded TBA to its unfolded structure was nearly constant regardless of EG concentration, suggesting that the free energies of water molecules around TBA do not strongly affect the stability of TBA.

**Figure 6.**
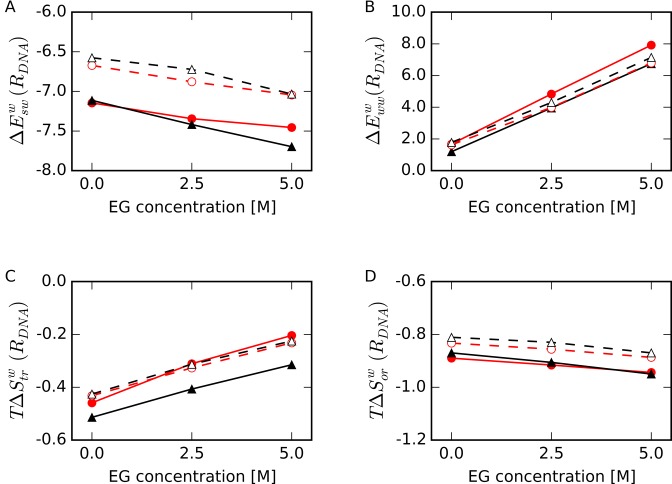
Normalized averages of thermodynamic parameters of a water molecule relative to bulk water in the first hydration shell around DNA (region *R_DNA_*) as a function of EG concentration. (**A**) Interaction energy between water and DNA, *ΔE_sw_^w^*(*R_DNA_*), (**B**) interaction energy between water molecules, *ΔE_ww_^w^*(*R_DNA_*), (**C**) translational entropy term, *TΔS_tr_^w^*(*R_DNA_*), and (**D**) orientational entropy term, *TΔS_or_^w^*(*R_DNA_*), for HP (filled red circles with solid lines), TBA (filled black triangles with solid lines), unHP (open red circles with dashed lines) and unTBA (open black triangles with dashed lines). All energies are shown in kcal/mol/water at 298 K.

To determine the dominant factor that contributes to the increase in *ΔG^w^*(*R_DNA_*) upon EG addition, we decomposed *ΔG^w^*(*R_DNA_*) into its components obtained by GIST (Figure 6). In the presence of EG, the solute-water interaction energy in *R_DNA_*, *ΔE_sw_^w^*(*R_DNA_*), was slightly decreased (∼0.5 kcal/mol/water at 298 K lower in 5.0 M EG) relative to that in the absence of EG (Figure 6A), whereas the difference in water–water interaction energy relative to bulk water in *R_DNA_*, *ΔE_ww_^w^*(*R_DNA_*), was largely increased (5–6 kcal/mol/water at 298 K higher in 5.0 M EG) (Figure 6B). The increase in *ΔE_ww_^w^*(*R_DNA_*) for HP was larger than those of other structures. The disruption in water–water interactions around HP appears to be caused by the intrusion of EG into the hydration shell, as shown in Figure 3B and 4B.

As shown in Figure 6C, in the presence of EG, the normalized average translational entropy term of one water molecule relative to bulk water in *R_DNA_*, *TΔS_tr_^w^*(*R_DNA_*) at 298 K, increased relative to that in the absence of EG for all structures, and the changes were correlated with *ρ_water_*(*R_DNA_*) as shown in Figure 4B. *TΔS_tr_^w^*(*R_DNA_*) for TBA was the lowest among all the structures. The normalized average orientational entropy term of one water molecule relative to bulk water in *R_DNA_*, *TΔS_or_^w^*(*R_DNA_*), was slightly decreased upon addition EG for all the structures (Figure 6D). *TΔS_or_^w^*(*R_DNA_*) values were lower than *TΔS_tr_^w^*(*R_DNA_*) values for all the structures and EG concentrations, and these values for folded structures were lower than those for unfolded structures. Thus, the orientation of water molecules around the folded DNA structures was strictly constrained regardless of EG concentration; however, the changes in solvation entropy terms, *TΔS_tr_^w^*(*R_DNA_*) and *TΔS_or_^w^*(*R_DNA_*), were smaller than those for the solvation interaction energies, *ΔE_sw_^w^*(*R_DNA_*) and *ΔE_ww_^w^*(*R_DNA_*). Among these parameters, changes in *ΔE_ww_^w^*(*R_DNA_*) upon EG were the largest, and the degree of change was largest for HP than for other structures. These results indicate that local structure and dynamics of water molecules around Watson–Crick base pairs are more readily disrupted by EG than are the water molecules around other structures.

### Local hydration properties around Watson–Crick base pairs and G-quartets

**Figure 7.**
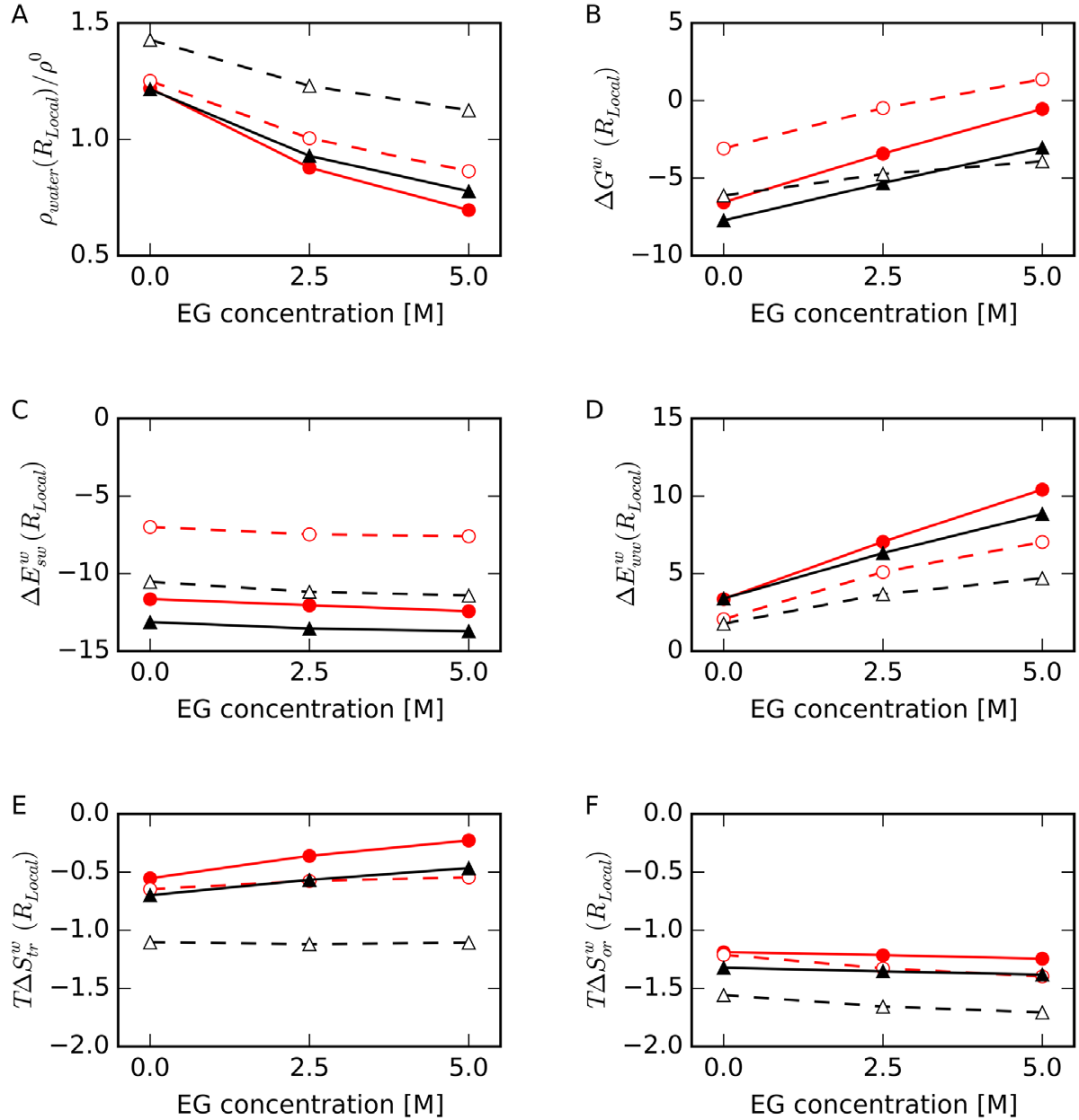
(**A**) Relative number density of water molecules, *ρ*(*R_Local_*)/*ρ*^0^, (**B**) normalized free energy of a water molecule, *ΔG^w^*(*R_Local_*), (**C**) normalized interaction energy between water and DNA, *ΔE_sw_^w^*(*R_Local_*), (**D**) normalized interaction energy between water molecules, *ΔE_ww_^w^*(*R_Local_*), (**E**) normalized translational entropy term, *TΔS_tr_^w^*(*R_Local_*) and (**F**) normalized orientational entropy term, *TΔS_or_^w^*(*R_Local_*), in *R_Local_* as a function of EG concentration for HP-backbone (filled red circles with solid lines), TBA-backbone (filled black triangles with solid lines), HP-base (open red circles with dashed lines) and TBA-base (open black triangles with dashed lines). Energies plotted in panels C, D, E and F are shown in kcal/mol/water at 298 K.

To evaluate local hydration properties in more detail, we focused on two Watson–Crick base pairs within HP (C4-G13 and T5-A12) and one G-quartet within TBA (involving G2, G5, G11 and G14) as shown in Figure 1A and B, respectively; these are internal pairs in order to eliminate contributions from loop and terminal regions. We will refer to the voxels around the base atoms in these nucleotides as HP-base and TBA-base and to the voxels around the phosphate groups as HP-backbone and TBA-backbone. The distances from the center of voxel to the designated atom of DNA were 2 Å < *r_min_* < 4 Å. We defined these regions as the local regions, *R_Local_*. Figure 7 shows the thermodynamic parameters of a water in the region *R_Local_* obtained by GIST analysis. The density of waters around base atoms was highest for TBA (*ρ_water_*(TBA-base)) as shown in Figure 7A, even though guanine bases are tightly packed in the quadruplex. In our definitions of *R_Local_* and *R_DNA_*, the inner area of TBA was excluded, because the region was too small to hold water molecules.

As shown in Figure 7B, *ΔG^w^*(HP-base) was the highest among all the local regions with or without EG due to the relatively high *ΔE_sw_^w^*(HP-base) values (Figure 7C). As shown in Figure 7C, *ΔE_sw_^w^*(TBA-base) was lower than *ΔE_sw_^w^*(HP-base), and *ΔE_ww_^w^*(TBA-base), *TΔS_tr_^w^*(TBA-base), and *TΔS_or_^w^*(TBA-base) were lowest among these values for all local regions as shown in Figure 7D, E and F, respectively. These results indicate that water molecules were more attracted to the bases of TBA than those of HP.

**Figure 8.**
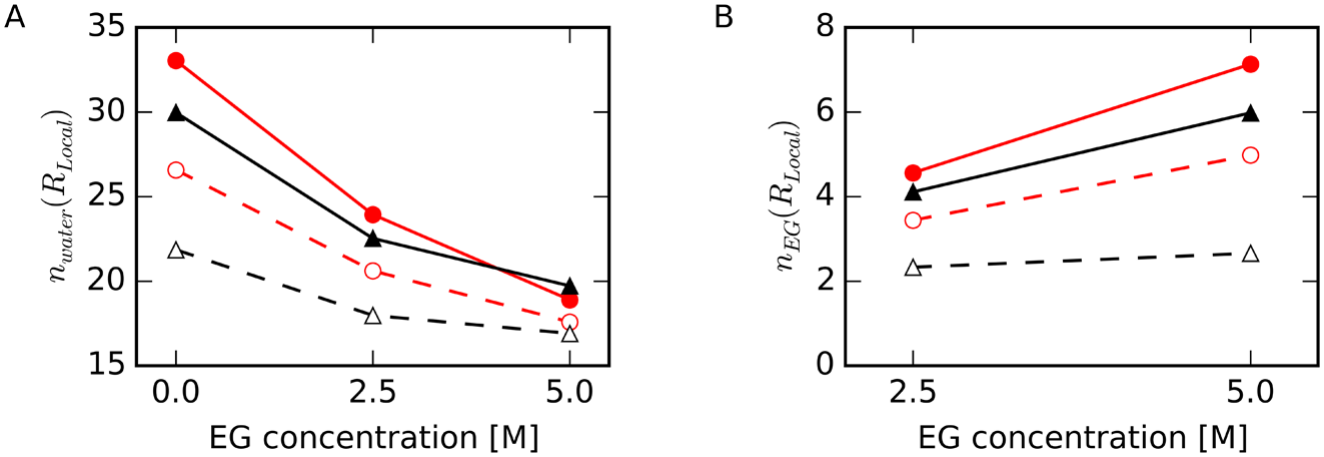
(**A**) Number of water molecules, *n_water_*(*R_Local_*), and (**B**) number of EG molecules, *n_EG_*(*R_Local_*), for HP-backbone (filled red circles with solid lines), TBA-backbone (filled black triangles with solid lines), HP-base (open red circles with dashed lines) and TBA-base (open black triangles with dashed lines).

Figure 8 shows the numbers of water molecules, *n_water_*(*R_Local_*), and EG molecules, *n_EG_*(*R_Local_*), in the local regions. Upon addition of EG, the number of waters in contact with the backbone atoms in HP, *n_water_*(HP-backbone), decreased and *n_EG_*(HP-backbone) increased most significantly, and *n_EG_*(TBA-base) changed the least. Changes in *n_water_* were consistent with changes in *ρ_water_* shown in Figure 7A. Our data indicate that EG more readily replaces water near Watson–Crick base pairs than around G-quartets.

**Figure 9.**
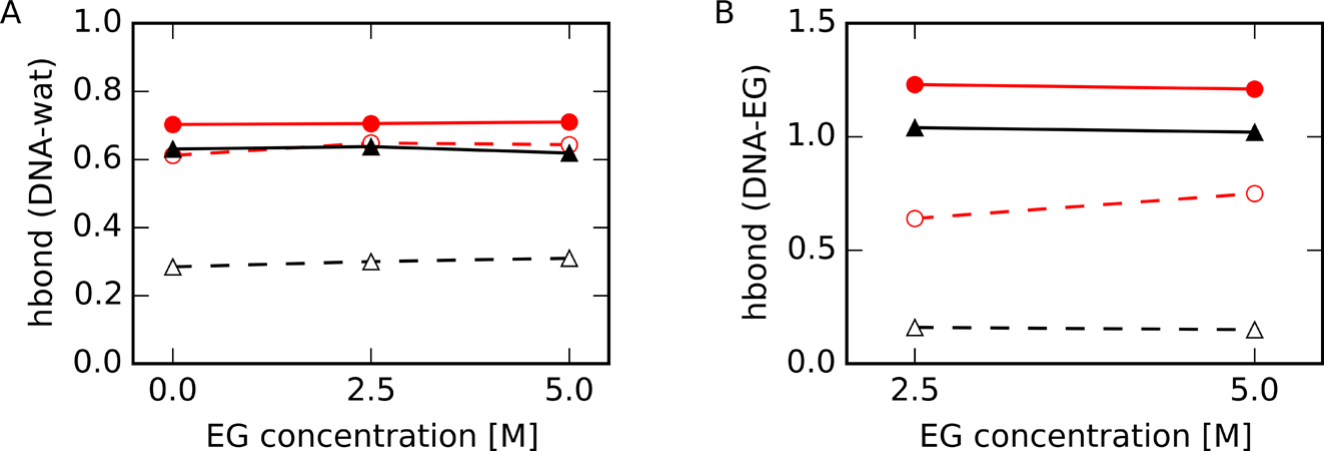
Number of hydrogen bonds between atoms within the specified region of DNA and (**A**) water or (**B**) EG for HP-backbone (filled red circles with solid lines), TBA-backbone (filled black triangles with solid lines), HP-base (open red circles with dashed lines) and TBA-base (open black triangles with dashed lines). Numbers of hydrogen bonds were normalized to the number of water or EG molecules in the region determined as shown in Figure 8.

We next counted the number of hydrogen bonds between water or EG molecules and the DNA atoms within the specified regions. We defined hydrogen bonds using the following criteria: the N–O or O–O distance was shorter than 3.5 Å and the N–H–O or O–H–O angle was between 135° and 180° (66). Numbers of hydrogen bonds were normalized by the number of water or EG molecules. As shown in Figure 9, the number of hydrogen bonds between EG and the atoms in HP-base increased with increasing EG concentration, although these numbers were not different between different EG concentration for other regions. These results are consistent with the results that more EG molecules contact HP than TBA, as shown in Figure 8B and in Figure 3B. On the other hand, the numbers of hydrogen bonds between a water molecule and the atoms in TBA-base were significantly lower than those in HP-base. This was expected since G(O6) and G(N7) are located inside the G-quartet, and these atoms are less accessible to water than are these atoms within Watson–Crick base pairs. These results appear to be inconsistent with the result that *ΔE_sw_^w^*(TBA-base) was lower than *ΔE_sw_^w^*(HP-base) (Figure 7C), as discussed in the next section.

## DISCUSSION

### G-quartets are more hydrated than Watson–Crick base pairs

There are inconsistencies in our data: *ΔE_sw_^w^* around the G-quartet were lower than that around Watson–Crick base pairs as shown in Figure 7C, whereas there were fewer hydrogen bonds per water molecule with the atoms in TBA than with the atoms in HP as shown in Figure 9A. We also found that the hydration shell around TBA was thicker than that around HP, although the peak heights of TBA were slightly lower than those of HP as shown in Figure 3A. These results indicate that water molecules interact with the atoms in G-quadruplex via non-hydrogen-bonded interactions, instead of forming hydrogen bonds with each atom of DNA.

TBA is a more globular structure than HP. It is possible that non-neutral globular TBA has a stronger electrostatic potential around it than the elongated molecule with the same net charge. If this is the case, TBA should attract more water molecules than HP. Therefore, we calculated the average electrostatic potential around the DNA, *V*(*r_min_*) and in the shell surface around DNA, *S_DNA_*(*r_min_*), as follows:

(11) \begin{equation*} V(r_{min} ) = \frac{1}{{N_P }}\left( {\sum\limits_{j \in S_{DNA} (r_{min} )}^{N_P } {\sum\limits_i^{N_{DNA} } {\frac{{q_i }}{{r_{ij} }}} } } \right). \end{equation*}

In Equation (11), *r_min_* is the minimum distance between any atoms in DNA and the shell surface around DNA, *S_DNA_*(*r_min_*), which is illustrated in Supplementary Figure S3A. *q_i_* is the atomic charge of DNA atom *i*, obtained from the force field parameters used in MD simulations, and *r_ij_* is the distance between DNA atom *i* and the point *j* located on *S_DNA_*(*r_min_*). *N_P_* is the number of calculated points on *S_DNA_*(*r_min_*), and *N_DNA_* is the number of DNA atoms.

**Figure 10.**
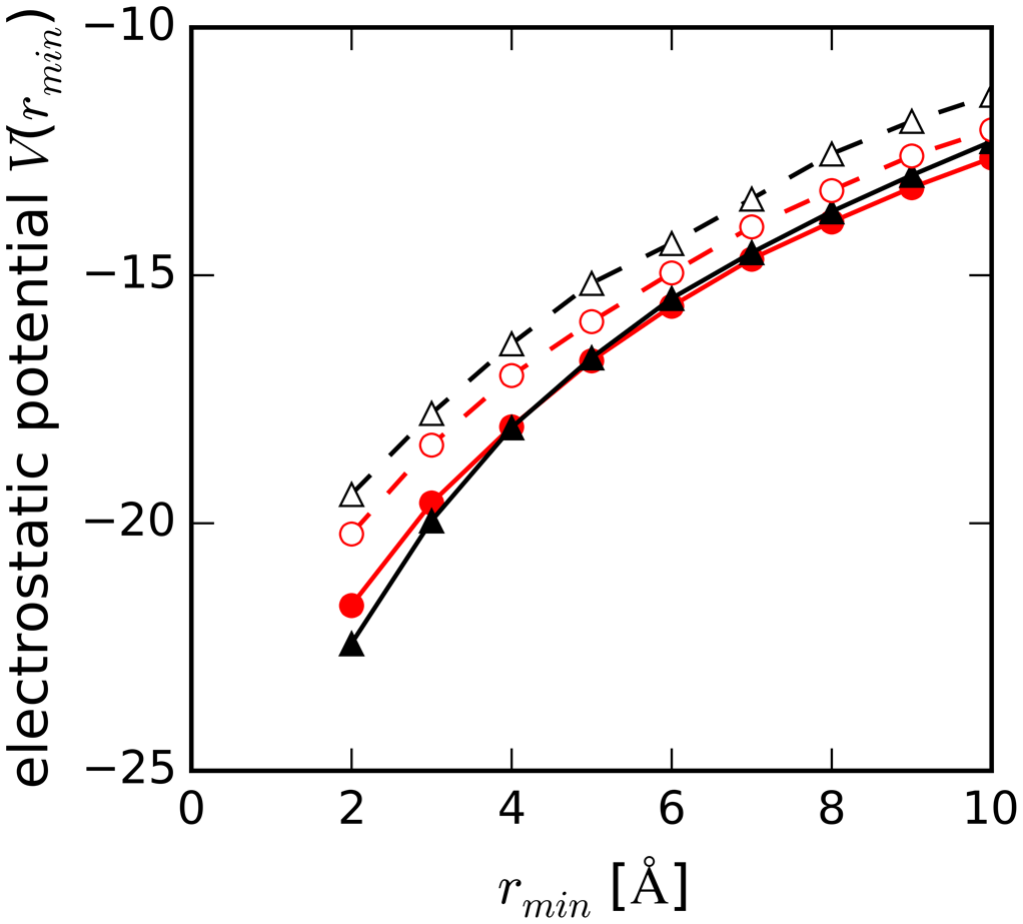
Averaged electrostatic potential (in arbitrary units) around DNA as a function of minimum distance from DNA, *r_min_*, for HP (filled red circles with solid line), TBA (filled black triangles with solid line), unHP (open red circles with dashed line) and unTBA (open black triangles with dashed line).

As shown in Figure 10, folded DNA structures created more negative electrostatic potentials, *V*(*r_min_*), around them than those around unfolded structures (*V*(*r_min_* = 2 Å) of −21.7 for HP, −22.4 for TBA, −20.2 for unHP and −19.4 for unTBA). *V*(*r_min_*) around unHP was more negative than that around unTBA regardless of *r_min_*, reflecting the difference in net charges of the molecules (−15 for unHP and −14 for unTBA). On the other hand, near the folded TBA (*r_min_* < 4 Å), *V*(*r_min_*) values were more negative than those around folded HP, although the net charge of HP was also more negative than that of TBA (−15 for HP and −14 for TBA). *V*(*r_min_*) values around HP and TBA were reversed at *r_min_* > 4 Å, and these values approached the values around their unfolded DNA structures at 20 Å from DNA. To confirm that the strong electrostatic potential around TBA was due to its structural geometry, we calculated the average distance distribution of the number of DNA atoms from the shell surface. The lengths from the shell surface in which all DNA atoms were included were 30 Å for TBA, 40 Å for HP, 50 Å for both unTBA and unHP (Supplementary Figure S3). As expected, the length for TBA was shortest among other structures.

The negative electrostatic potential is expected to attract water molecules. Because we counted the oxygen atoms of water in order to calculate the water density around DNA, water density distributions around DNAs as shown in Figure 3A were consistent with the distribution of electrostatic potential around DNAs: the peak heights of the water density at *r_min_* of 2.5 Å around TBA were smaller than that around HP, and the densities from 3.0 Å to 4.0 Å around TBA were larger than those around HP.

Since G(O6) and G(N7) are hydrogen bonded in a G-quartet, fewer base atoms in the G-quartet were able to form hydrogen bonds with water molecules than in Watson–Crick base pairs. Water molecules form cooperative hydrogen bonding networks with DNA atoms in the minor groove of double-stranded DNA (67,68). The numbers of hydrogen bonds observed were associated with these geometrical differences (Figure 9). Our results demonstrated that the negative electrostatic potential around the G-quadruplex contributed to formation of a thick hydration shell. These water molecules were not easily disrupted by EG and had lower solvation entropy terms and solvation interaction energies compared to water molecules associated with the hairpin and single-stranded structures as shown in Figures 6 and 7.

### EG easily replaces water molecules around Watson–Crick base pairs

Another new finding in this study is the preferential binding of EG to HP. Fewer EG molecules were held around TBA than around HP (Figure 3B), and EG formed very few hydrogen bonds with G-quartet bases (Figure 9B). In addition, the number of hydrogen bonds between EG and the atoms in G-quartet did not increase with increasing EG concentration. To investigate why EG molecules more easily access the surface of HP than that of TBA, we calculated the relative solvent accessible surface areas (*R_SASA_*) of DNAs as a function of a spherical probe of radius, *r_p_*:

(12) \begin{equation*} R_{SASA} = \frac{{SASA(r_p )}}{{SASA(r_0 )}}. \end{equation*}

**Figure 11.**
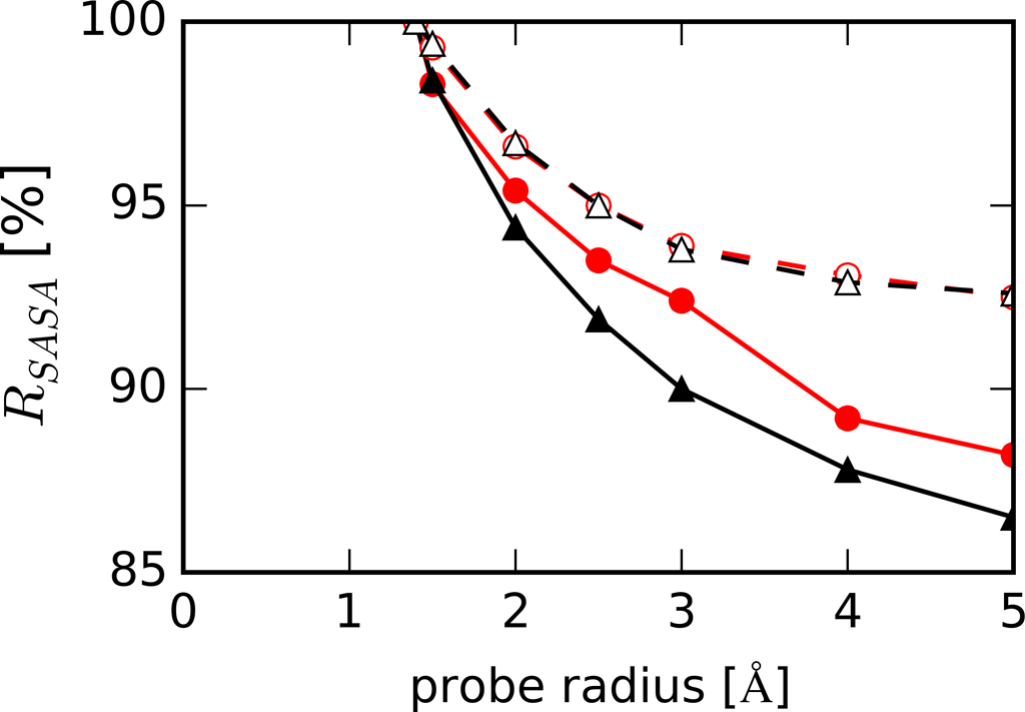
Ratio of solvent accessible surface areas, *R_SASA_*, of DNAs measured as a function of probe radius (*r_p_*) for HP (filled red circles with solid line), TBA (filled black triangles with solid line), unHP (open red circles with dashed line) and unTBA (open black triangles with dashed line).

In Equation (12), *SASA*(*r_p_*) and *SASA*(*r_0_*) are the solvent accessible surface areas measured with probe radius *r_p_* and *r_0_*. *r_0_* is the referenced probe radius, set to be 1.4 Å for this calculation; this value is often used as the probe radius of a water molecule for the calculation of solvent accessible surface area (69,70). As shown in Figure 11, *R_SASA_* for TBA decreased more significantly with increasing *r_p_* than those for other structures. The gyration radius of EG was about 2.4 times that of water, which were calculated by cpptraj module in Amber14 (64). These results indicated that the pocket sizes on the surface of TBA were relatively small for large cosolute, such as EG, but large enough for small molecule, such as water, to bind. As a result, EG could easily access the surface of HP compared to the surface of TBA.

Nordstrom *et al*. (71) showed using the local-bulk partitioning model that EG locally accumulates at DNA strands independently of base identity. Our calculations showed that the HP surface was more accessible to EG than was the surface of unfolded DNA (Figure 3B and D). Furthermore, EG was attracted to the backbone region of Watson–Crick base pairs rather than the bases (Figure 8B). These results are consistent with the data from Nordstrom *et al*., which indicated that EG was more often found around the phosphate groups of a DNA duplex than around the bases. Water–water interactions around HP were disturbed more significantly by the cosolutes than were those around TBA. In addition, the free energy of water molecules around the unfolded structure was lower than that around the folded HP structure at the high EG concentration as shown in Figure 5B, and this free energy difference likely contributes to difference in the equilibrium between HP and unHP in the presence and absence of EG.

### Free energy of the water around DNA atoms contributes to the thermal stability of DNA structures

In this study, we focused on the thermodynamic parameters of water molecules around DNA. Cosolutes in the solvent affect not only the water activity of a system but also the free energy of water molecules around DNA. In Table 2, we summarize the differences in thermodynamic parameters of water molecules in the first hydration shells around each DNA structure in the presence and absence of EG. Dominant factor on the increasing of free energy of water molecules around the solute caused by cosolutes was the disturbed water–water interaction. Generally, attractive solute–water interactions result in unfavorable water–water interactions (42). However, our results showed that *ΔE_sw_^w^* decreased slightly whereas *ΔE_ww_^w^* increased in the presence of EG relative to its absence. This may be because *ΔE_ww_^w^* is governed by the orientations of the water molecules around DNA, whereas *ΔE_sw_^w^* is determined by the relationship between DNA and each water molecule. The effect of cosolute on the free energy of water molecules, *Δ*[*ΔG^w^*(*R_DNA_*)] around HP was the highest among structures analyzed. This is consistent with the result that the thermal stability of HP decreased in the presence of cosolutes, whereas that of TBA did not (26–28). In this study, there was no difference in *Δ*[*ΔG^w^*(*R_DNA_*)] between TBA and unTBA. This result does not contradict the experimental result that the cosolutes increased the thermal stability of TBA since factors not addressed in this study, such as free energy of cosolute and free energy differences among different unfolded DNA structures, likely affect this equilibrium. Our results indicate that the disruption of water–water interactions caused by cosolutes would affect the system equilibrium.

**Table 2.** Differences in thermodynamic parameters (in kcal/mol/water at 298 K) of water molecule in the first hydration shell around DNA, *R_DNA_*, in the presence and absence of EG^a^

Name	Δ[TΔS_tr_^w^(R_DNA_)]	Δ[TΔS_or_^w^(R_DNA_)]	Δ[ΔE_sw_^w^(R_DNA_)]	Δ[ΔE_ww_^w^(R_DNA_)]	Δ[ΔG^w^(R_DNA_)]
HP	^b^ 0.24 ± 0.005	−0.06 ± 0.007	−0.30 ± 0.060	6.25 ± 0.091	5.77 ± 0.123
TBA	0.19 ± 0.004	−0.09 ± 0.004	−0.58 ± 0.055	5.56 ± 0.170	4.88 ± 0.175
unHP	0.18 ± 0.003	−0.06 ± 0.008	−0.37 ± 0.069	5.17 ± 0.093	4.68 ± 0.116
unTBA	0.18 ± 0.002	−0.07 ± 0.003	−0.46 ± 0.044	5.36 ± 0.064	4.79 ± 0.067

^a^Δ[ΔQ(R_DNA_)] = [ΔQ(R_DNA_)]_EG = 5.0M_ − [ΔQ(R_DNA_)]_EG = 0.0M_.

^b^Standard errors of the mean (SEM) were calculated with the average values for every 10 ns data from 50 ns to 100 ns MD simulations.

Hammouda and Worcester investigated DNA denaturation transition in water and EG mixed solvents using ultraviolet light absorption spectroscopy and small-angle neutron scattering (72). They demonstrated that DNA melting transition temperature was decreased linearly with the increasing of EG concentration. They also demonstrated that in the mixture solvent, solvation shell around unfolded DNA structure has less-stress compared to that around the DNA helix. They suggest that mixed solvents tend to arrange themselves efficiently so as to minimize conformational ‘stress’ around the polymer coils in the solvation shell. Their findings are consistent with our results showing that water molecules around Watson–Crick base pairs are unstable compared to those around unfolded DNA structure.

### DNA strands do not take up water during the formation of Watson–Crick base pairs

Many previous studies using osmotic stress measurements with different analytical instrumentation have demonstrated that the logarithm of *K_obs_* is proportional to the logarithm of *a_water_*, where *K_obs_* is the observed thermodynamic equilibrium constant between folded and unfolded DNA structures and *a_water_* is the water activity regulated by cosolutes (23,27–29,33–37,71). In these studies, ∂(ln *K_obs_*) / ∂(ln *a_water_*) was interpreted as the difference in the number of water molecules bound to DNA strands, Δ*n_water_*, in folded and unfolded DNA structures. The incline of the slope showed that water molecules are taken up by DNA from bulk water during the formation of Watson–Crick base pairs, and the decline of the slope showed that water molecules are released from DNA strands during the formation of G-quadruplexes with Hoogsteen base pairs (23,27,33,35).

This interpretation seems to conflict the microscopic picture that the solvent accessible surface area around the Watson–Crick base pair is smaller than the sum of that around the isolated bases (38). Our results showed that upon formation of Watson–Crick base pairs and G-quartets, dehydration occurred (Figure 4A), although more water molecules were released upon formation of a G-quadruplex than a DNA duplex. Recently, Son *et al*. demonstrated using ultrasonic velocimetric and densimetric measurements that DNA duplex association is accompanied by the release of water molecules from the hydration shell (38). It should be noted that our unfolded DNA structures do not reflect the flexibility of the single-stranded state in solution. In addition, we only focused on the water molecules in the first hydration shell because we did not observed significant differences in water densities further from the DNA atoms, and the hydration volume around DNAs is expected to be governed by the solvent accessible surface area. Even considering our calculation conditions were insufficient, results of our calculation are consistent with the results of Son's experiment. Son *et al*. also showed that cosolute can alter the number of hydrated waters around a DNA and the water activity. Our results also demonstrated that the number of water molecules around a DNA decreased with increasing EG concentration (Figures 4 and 8).

It has been shown that the formation of Watson–Crick base pairs is unfavorable in solvent with low water activity (23,27–29,33–37). This result indicates that the presence of water molecules is essential for the formation of Watson–Crick base pairs. The affinity of water molecules for DNA should depend on the binding position on the DNAs, and water molecules bound at different locations on a DNA structure will not have equivalent impacts on the thermal stability of a structure. The numbers of waters bound to folded and unfolded states determined in the cited studies are averages of thermodynamically relevant water molecules in all hydration shells; these values do not reflect the number of water molecules immediately surrounding the DNAs analyzed in the present study. The slope of ∂(ln *K_obs_*) / ∂(ln *a_water_*) may be closely correlated with the role of water molecule in the thermodynamic stability of DNA structures, but it is not simply the total number of water molecules around the DNA.

Here, using MD simulations and GIST, we studied the molecular mechanism of how cosolutes affect the thermal stabilities of DNA structures. We propose that disruption of water–water interactions around DNA by cosolutes results in structure-dependent impacts of cosolute on the thermal stability of DNA structures. At high EG concentration, water molecules around the unfolded DNA structure were more stable thermodynamically than those around Watson–Crick base pairs, whereas there was no significant difference in thermodynamic properties of water molecules with folded G-quadruplex and the unfolded structure. These free energies of water molecules around folded and unfolded DNA would contribute to the equilibrium between them. Our results suggest that the free energy for the system including solvents and cosolutes should be considered when investigating the thermodynamic equilibrium state of DNA structures. The unfolded DNA structure used in this study is the only one structure among the numerous configurations which the single stranded DNA can adopt in aqueous environment (52–55). The hydration properties around unfolded DNA strand likely depend on the unfolded configurations. To further understand how the thermal stability of DNA is affected by solvent, including abundant cosolutes, we must take into account the entropy of single-stranded DNA and the free energy of cosolutes as well as water molecules around DNA. In addition, the interaction among cosolutes, DNA, and water molecules also should be considered. These issues will be addressed in the future work.

## Supplementary Material

SUPPLEMENTARY DATA
