# Supplementary material for: Thermodynamic properties of water molecules in the presence of cosolute depend on DNA structure: a study using grid inhomogeneous solvation theory
Source: Nucleic Acids Res. 2015 Nov 3;43(21):10114–25. doi: 10.1093/nar/gkv1133 (PMC4666364; doi:10.1093/nar/gkv1133)
Supplement: SUPPLEMENTARY DATA [file supp_43_21_10114__index.html]

Thermodynamic properties of water molecules in the presence of cosolute depend on DNA structure: a study using grid inhomogeneous solvation theory — SUPPLEMENTARY DATA 

# Thermodynamic properties of water molecules in the presence of cosolute depend on DNA structure: a study using grid inhomogeneous solvation theory

## SUPPLEMENTARY DATA

- SUPPLEMENTARY DATA
