# Supplementary material for: Thermodynamic properties of water molecules in the presence of cosolute depend on DNA structure: a study using grid inhomogeneous solvation theory
Source: Nucleic Acids Res. 2015 Nov 3;43(21):10114–25. doi: 10.1093/nar/gkv1133 (PMC4666364; doi:10.1093/nar/gkv1133)
Supplement: SUPPLEMENTARY DATA [file supp_gkv1133_nar-02276-f-2015-File001.pdf]

Thermodynamic Properties of Water Molecules in the Presence of Cosolute Depend on DNA Structure: A Study using Grid Inhomogeneous Solvation Theory

Miki Nakano[1,2], Hisae Tateishi-Karimata[1], Shigenori Tanaka[3], Florence Tama[2,4], Osamu Miyashita[2], Shu-ichi Nakano[5] & Naoki Sugimoto[1,6*]

[1] Frontier Institute for Biomolecular Engineering Research (FIBER), Konan University, 7-1-20 Minatojima-minamimachi, Chuo-ku, Kobe 650-0047, Japan.
[2] Advanced Institute for Computational Sciences, RIKEN, 7-1-26, Minatojima-minamimachi, Chuo-ku, Kobe, 650-0047, Japan
[3] Department of Computational Science, Graduate School of System Informatics, Kobe University, 1-1, Rokkodai, Nada-ku, Kobe, 657-8501, Japan
[4] Department of Physics, Graduate School of Science, Nagoya University, Furo-cho, Chikusa-ku, Nagoya, 464-8602, Japan
[5] Faculty of Frontiers of Innovative Research in Science and Technology Konan University, 7-1-20, Minatojima-minamimachi, Chuo-ku, Kobe, 650-0047, Japan
[6] Graduate School of Frontiers of Innovative Research in Science and Technology (FIRST), Konan University, 7-1-20 Minatojima-minamimachi, Chuo-ku, Kobe 650-0047, Japan.

* To whom correspondence should be addressed. Tel: +81-78-303-1416; Fax: +81-78-303-1495; Email: sugimoto@konan-u.ac.jp

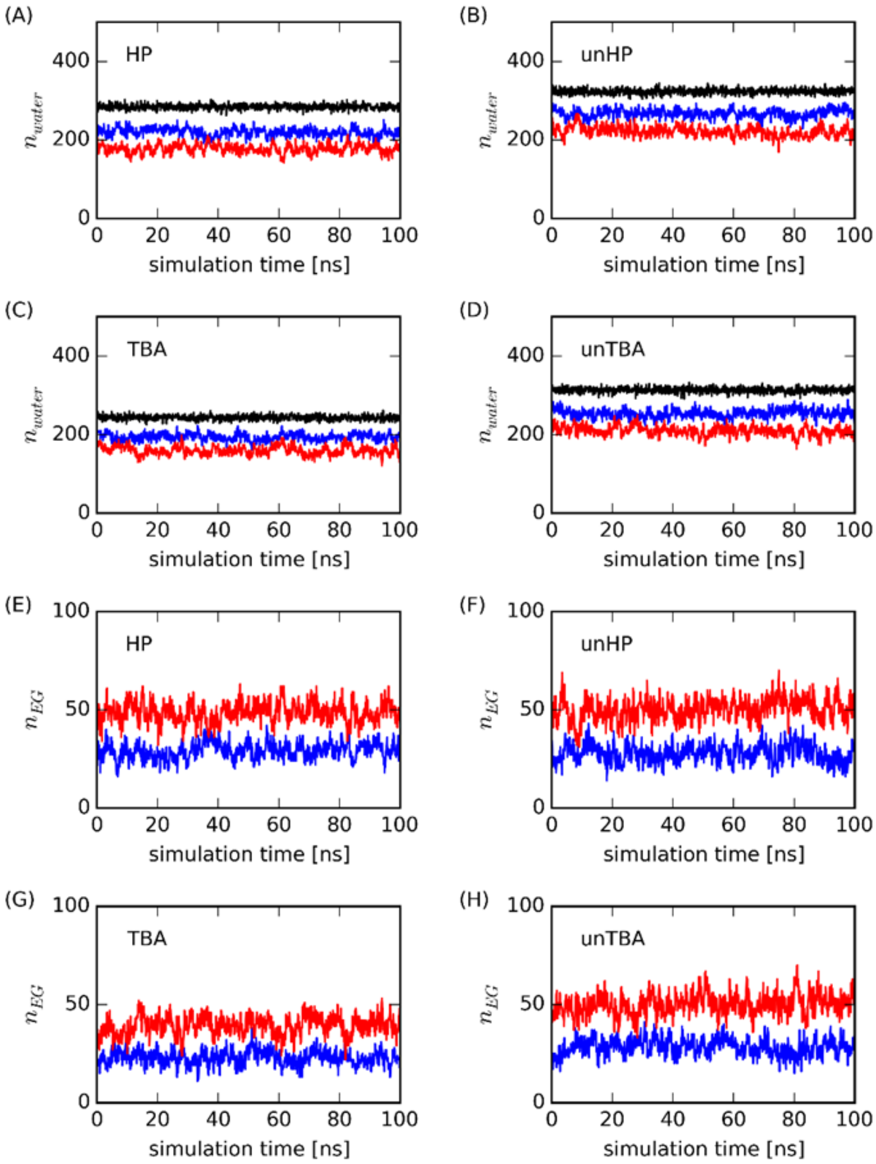

Supplemental Figure 1. Number of water molecules, n_{water}, within 4.0 Å of atoms of (**A**) HP, (**B**) unHP, (**C**) TBA, and (**D**) unTBA. Number of EG molecules, n_{EG}, within 4.0 Å of atoms of (**E**) HP, (**F**) unHP, (**G**) TBA, and (**H**) unTBA. Black, blue, and red lines represent for 0.0 M, 2.5 M, and 5.0 M EG concentrations, respectively.

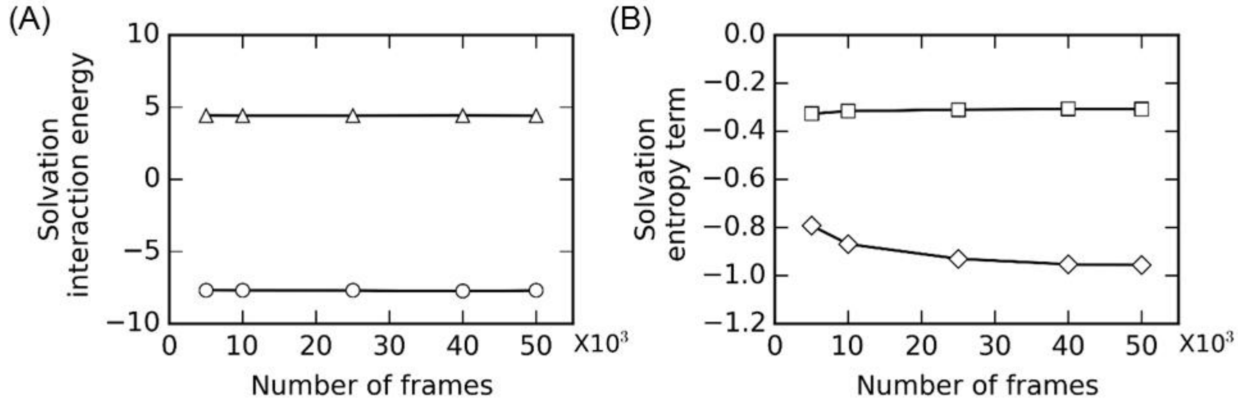

Supplemental Figure 2. Convergence of thermodynamic parameters for a water molecule in the region R_{DNA} around TBA for 5.0 M EG concentration using different number of frames. The definition of R_{DNA} is described in the main text. (A) Solvation interaction energies $\Delta E_{sw}{}^{w}(R_{DNA})$ and $\Delta E_{ww}{}^{w}(R_{DNA})$, represented by circles and triangles, respectively, as a function of the number of frames. (B) Solvation entropies $T\Delta S_{tr}{}^{w}(R_{DNA})$ and $T\Delta S_{or}{}^{w}(R_{DNA})$, represented by squares and diamonds, respectively, as a function of the number of frames. All energies are shown in kcal/mol/water at 298 K.

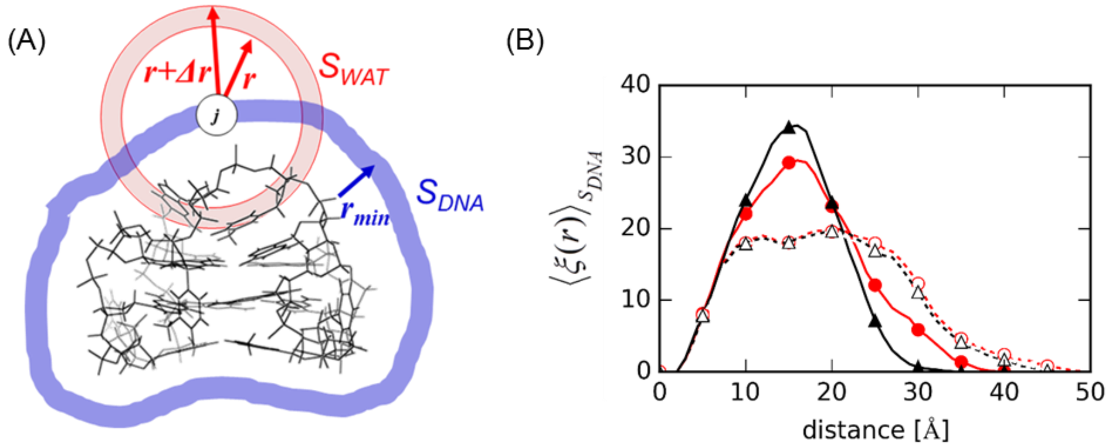

Supplemental Figure 3. (A) Schematic view of the shell surface around DNA, $S_{DNA}(r_{min})$, used in eq. (11) in main text. r_{min} is the minimum distance between any atoms in DNA and S_{DNA}. (B) The average distance distribution of the number of DNA atoms, $\langle \xi(r) \rangle_{S_{DNA}}$, from the shell surface S_{DNA}(3 Å < r_{min} < 4 Å) around each DNA structure for HP (filled red circles with solid lines), TBA (filled black triangles with solid lines), unHP (open red circles with dashed lines), and unTBA (open black triangles with dashed lines). $\langle \xi(r) \rangle_{S_{DNA}}$ refers to the average number of DNA atoms within the spherical shell with the radius $r \sim r+\Delta r$, S_{WAT}: illustrated in panel A, with the center is located within S_{DNA}, and defined as follows: $\langle \xi(r) \rangle_{S_{DNA}} = \frac{1}{N_P} \sum_{j \in S_{DNA}(r_{\min})}^{N_P} \int_r^{r+\Delta r} \sum_{i=1}^{N_{DNA}} \delta(r' - r_{ij}) dr'$, r_{ij} is the distance between DNA atom i and the point j within S_{DNA}. N_P is the number of sample points within S_{DNA}, and N_{DNA} is the number of DNA atoms.
